# Factors Associated With In-hospital Mortality of Children With Acute Fulminant Myocarditis on Extracorporeal Membrane Oxygenation

**DOI:** 10.3389/fped.2020.00488

**Published:** 2020-08-27

**Authors:** En-Pei Lee, Sheng-Chih Chu, Wun-Yan Huang, Shao-Hsuan Hsia, Oi-Wa Chan, Chia-Ying Lin, Ya-Ting Su, Yu-Sheng Chang, Hung-Tao Chung, Han-Ping Wu, Jainn-Jim Lin

**Affiliations:** ^1^Division of Pediatric Critical Care Medicine, and Pediatric Sepsis Study Group, Chang Gung Children's Hospital and Chang Gung Memorial Hospital, Chang Gung University College of Medicine, Taoyuan, Taiwan; ^2^College of Medicine, Chang Gung University, Taoyuan, Taiwan; ^3^Division of Pediatric Cardiovascular Internal Medicine, Chang Gung Children's Hospital and Chang Gung Memorial Hospital, Chang Gung University College of Medicine, Taoyuan, Taiwan; ^4^Department of Pediatrics, Taoyuan Armed Forces General Hospital, Taoyuan, Taiwan; ^5^National Defense Medical Center, Taoyuan, Taiwan; ^6^Department of Pediatric Emergency Medicine, China Medical University Children Hospital, Taichung, Taiwan; ^7^Department of Medicine, School of Medicine, China Medical University, Taichung, Taiwan; ^8^Department of Medical Research, Children's Hospital, China Medical University, Taichung, Taiwan; ^9^Division of Cardiovascular Surgery, Chang Gung Children's Hospital and Chang Gung Memorial Hospital, Chang Gung University College of Medicine, Taoyuan, Taiwan

**Keywords:** acute fulminant myocarditis, predictors, mortality, children, VA-ECMO

## Abstract

**Aim:** To analyze the factors associated with in-hospital mortality of children with acute fulminant myocarditis on venoarterial extracorporeal membrane oxygenation (VA-ECMO).

**Methods:** This was a retrospective cohort study using chart reviews of patients diagnosed with acute fulminant myocarditis at the pediatric intensive care unit of two tertiary medical centers between January 1, 2005 and December 31, 2017. The inclusion criteria for this study were: (1) age from 1 month to 18 years; (2) diagnosed with acute myocarditis; (3) cardiogenic shock and need vasoactive-inotropic score ≥20 within 48 h after the use of vasoactive-inotropic agents; and (4) the need for ECMO placement.

**Results:** Thirty-three children with acute fulminant myocarditis who needed ECMO were included. Clinical parameters were retrospectively reviewed. The overall survival rate was 69.6%. Higher levels of pre-ECMO troponin-I and pre-ECMO lactate, and lower post-ECMO left ventricular ejection fraction (LVEF) were significantly associated with in-hospital mortality in univariate analysis. Only higher pre-ECMO lactate and lower post-ECMO LVEF remained as predictors for in-hospital mortality in multivariate analysis. The areas under the curve of pre-ECMO lactate and post-ECMO LVEF in predicting survival were 0.848 (95% CI, 0.697–0.999, *p* = 0.002) and 0.824 (95% CI, 0.704–0.996, *p* = 0.01), respectively. A pre-ECMO lactate level of 79.8 mg/dL and post-ECMO LVEF of 39% were appropriate cutoff points to predict mortality.

**Conclusion:** Pre-ECMO lactate level was associated with mortality in children with acute fulminant myocarditis, with an optimal cutoff value of 79.8 mg/dL. After VA-ECMO implantation, post-ECMO LVEF was associated with mortality, with an optimal cutoff value of 39%. The use of LVADs or urgent heart transplantation should be considered if the post-ECMO LVEF does not improve.

## Introduction

Acute myocarditis is an inflammatory disease of heart muscles (1), and it is caused by many etiologies such as infection, autoimmune dysregulation, hypersensitivity reactions, and some toxins. Acute myocarditis is uncommon in children, with an estimated annual incidence of 1–2 per 100,000 children (2, 3) with a bimodal age distribution (infancy and adolescence) (4, 5). The clinical manifestations range from minor symptoms to severe heart failure or sudden death. Due to the variable presentations of pediatric myocarditis, making a correct timely diagnosis is not easy, and misdiagnosis as pneumonia, bronchiolitis, or acute gastroenteritis is not uncommon (5, 6). Furthermore, previous studies have reported a high incidence of myocarditis (10–20%) confirmed by autopsy in children who experience sudden death (7–11). Therefore, it is important to recognize the early symptoms and initiate appropriate therapy, as this may improve the outcomes.

The main therapeutic strategy for acute myocarditis remains supportive to maintain stable hemodynamics and sufficient organ perfusion. However, rapidly progressive heart failure in children indicates acute fulminant myocarditis, and venoarterial extracorporeal membrane oxygenation (VA-ECMO) is recommended for persistent lactic acidosis and poor perfusion of end organs despite pharmacological treatment (12). The use of VA-ECMO support has increased in recent years, and around 20% of hospitalized pediatric patients with acute fulminant myocarditis in America receive VA-ECMO (4). VA-ECMO supports cardiopulmonary function, providing blood and oxygen for vital organs and allowing the heart and lungs to rest. However, VA-ECMO cannot rescue all patients, and around 40% cannot be successfully weaned from VA-ECMO and subsequently die (4, 13). Factors associated with mortality after VA-ECMO include persistent arrhythmia, the need for dialysis, and end-organ hypoperfusion (12, 13). However, few studies have investigated the factors associated with in-hospital mortality of children with acute fulminant myocarditis on ECMO. Therefore, the aim of this study was to compare the clinical factors pre-ECMO and after VA-ECMO implantation between survivors and non-survivors and to identify the factors associated with in-hospital mortality in children with acute fulminant myocarditis.

## Materials and Methods

### Patient Population

This was a retrospective cohort study using chart reviews of patients diagnosed with acute fulminant myocarditis at the pediatric intensive care unit of two tertiary medical centers (Chang Gung Children's Hospital and China Medical University Children Hospital) between January 1, 2005 and December 31, 2017. The inclusion criteria for this study were: (1) age from 1 month to 18 years; (2) diagnosed with acute myocarditis; (3) cardiogenic shock and need vasoactive-inotropic score ≥20 within 48 h after the use of vasoactive-inotropic agents; and (4) the need for ECMO placement. The exclusion criteria were: (1) those older than 18 years; (2) vasoactive-inotropic score ≤20; and (3) those who did not receive ECMO placement. This study was approved by the Institutional Review Board of Chang Gung Memorial Hospital (IRB Number: 201800094B0).

### Definitions

The diagnosis of acute myocarditis in our study were based on the clinical diagnosis which met the criteria of probable acute myocarditis cited in two representative journals (The lancet 2012 and Circulation 2014). The clinical diagnosis were based on clinical histories, abnormal electrocardiographic findings (ST elevation, T inversion or conduction block), left ventricular dysfunction evaluated by cardiac sonography, laboratory data (i.e., cardiac enzyme, C-reactive protein), and a recent history of viral infection (1, 14, 15). In the two hospitals, endomyocardial biopsies (EMB) are not performed for acute fulminant myocarditis in children due to their critical condition (16). Vasoactive-inotropic score was defined as dopamine dose (mcg/kg/min) + dobutamine dose (mcg/kg/min) + 10 × milrinone dose (mcg/kg/min) + 100 × epinephrine dose (mcg/kg/min) + 100 × norepinephrine dose (mcg/kg/min) + 10,000 × vasopressin dose (units/kg/min). A vasoactive-inotropic score more than 20 indicated severe impairment of cardiovascular system (17). VA-ECMO was implanted due to persistent unstable hemodynamics under high dose of vasoactive-inotropic agents, refractory ventricular arrhythmia, or who experienced cardiopulmonary resuscitation (18).

### Details of Support

The VA mode of ECMO involved the insertion of arterial and venous cannulas to vessels in the neck and inguinal area or directly to the aorta and right atrium. The ECMO system used in our hospital includes a centrifugal pump [Capiox emergent bypass system (Terumo, Tokyo, Japan) or Bio-console 560 system (Medtronic, Minneapolis, MN, USA)], a hollow-fiber microporous membrane oxygenator (Medtronic, Minneapolis, MN, USA), an integrated heat exchanger, and an oxygen blender. Heparin given intravenously was used as an anti-coagulant to prevent microthrombi formation (keep activated clotting time: 180–220 s) along the inner surface of the ECMO circuit. The flow rate of ECMO in VA mode was initially set at ~40–100 c.c/kg/min to keep the mean arterial pressure above 50 mmHg. We administered midazolam, ketamine, and cisatracurium to sedate and paralyze the patients if necessary. Intravenous heparin was administered to control the activated clotting time to around 160–200 s. Daily cardiac sonography was performed, and weaning from ECMO was attempted by slowing down the pump if improvements in cardiac contractility and pulmonary function were noted (19, 20).

### Data Collection

The following information was collected for all patients: (1) demographic data; (2) hemodynamic condition and cardiac rhythm; (3) laboratory examinations before and after ECMO; (4) the incidence of acute kidney injury and the need for hemodialysis; and (5) outcomes including the duration of ECMO and the days of hospitalization.

### Statistical Analysis

The patients were divided into two groups: survival and non-survival group. The patient characteristics in each study group are presented as descriptive statistics, and the data are presented as percentage (%) or median (interquartile range [IQR]). For comparisons of dichotomous variables between groups, the chi-square test or Fisher's exact test was used. Comparisons of continuous variables between the two groups were performed using the Mann–Whitney *U*-test. Predicted probabilities of mortality and 95% confidence intervals (CIs) were calculated using a Cox regression model. Finally, receiver operating characteristic (ROC) curves were used to determine the ideal cutoff values for the independent predictors of mortality. The test characteristics of the different cutoff values, including sensitivity, specificity, area under the ROC curve (AUC), positive likelihood ratio (LR+), and negative likelihood ratio (LR–) were also examined. Statistical significance was set at *p* < 0.05. All statistical analyses were performed using SPSS software (version 22.0; SPSS Inc., Chicago, IL, USA).

## Results

### Patients

During the study period, 33 children with acute fulminant myocarditis who needed ECMO placement were included. Before the initiation of VA-ECMO, all children received mechanical ventilation and at least two vasoactive-inotropic agents. However, all of the included children experienced a deterioration in cardiac ventricle function (cardiogenic shock) accompanied by multi-organ dysfunction or cardiac arrest which prompted the initiation of VA-ECMO. The presumed pathogens of acute fulminant myocarditis are reported in Table 1, in which unknown pathogens accounted for the majority in both survival and non-survival groups.

**Table 1:** Presumed pathogens in 33 children with acute fulminant myocarditis.

**Survivors**	*n* = 23 (69.6%)
Coxsackie B	*n* = 3
Enterovirus 71	*n* = 3
Influenza type B	*n* = 2
Parvovirus B19	*n* = 2
Human herpesvirus 6	*n* = 1
Unknown	*n* = 12
**Non-survivors**	*n* = 10 (30.4%)
Enterovirus 71	*n* = 3
Mycoplasma pneumonia	*n* = 1
Influenza type B	*n* = 1
Human herpesvirus 6	*n* = 1
Unknown	*n* = 4

### Comparisons Between the Survivors and Non-survivors

The age annulation was shown in Table 2 that the median age in the survival group was 8.2 and 8.6 years in the non-survival groups. During the in-hospital period, 23 children survived and 10 children died (survival rate 69.6%). There were no significant differences in general demographics including age, sex, weight, and PRISM III score between the survival and non-survival groups (Table 2). There were no significant differences in the initial hemodynamic condition, except that the non-survival group had a lower post-ECMO left ventricular ejection fraction (LVEF) (*p* = 0.001). With regards to the biochemistry and physiologic data, the non-survival group had significantly higher levels of pre-ECMO lactate and post-ECMO peak troponin-I (*p* < 0.05). The median ECMO duration was 120 h (IQR 95–201 h) in the survival group and 134 h (IQR 67–187 h) in the non-survival group (*p* = 0.985). The median duration of hospitalization was 26 days (IQR 21–57 days) in the survival group and 8 days (IQR 6–12 days) in the non-survival group (*p* = 0.001).

**Table 2:** Comparisons between survivors and non-survivors.

	**Survivors (*n* = 23)**	**Non-survivors (*n* = 10)**	***p***
**General demographics**			
Age (years), median (IQR)	8.2 (6.2–13.5)	8.6 (3.2–11.3)	0.857
Male (%)	10 (43.4)	3 (30)	0.482
Weight (kg), median (IQR)	27 (20–40)	27 (12–40)	0.893
PRISM III score, median (IQR)	25 (18–30)	34 (21–37)	0.057
**Cardiac rhythm**			
AV block	4 (17.3)	2 (20)	0.862
VT	10 (43.4)	6 (60)	0.383
PSVT	1 (4)	0	0.5
**Hemodynamic condition, median (IQR)**			
ECPR (%)	3 (13)	4 (40)	0.08
SBP (mmHg)	92 (76–106)	86 (60–103)	0.603
DBP (mmHg)	53 (39–67)	49 (41–64)	0.985
Vasoactive-inotropic score	34 (21–39)	39 (29–62)	0.144
Pre-ECMO LVEF (%)	38 (28–45)	33 (19.5–40)	0.287
Post-ECMO LVEF (%)	56 (44-62)	34.6 (28-42)	0.001^*^
**Laboratory examination, median (IQR)**			
**Pre-ECMO**			
Hemoglobin (g/dL)	11 (8.9–11.9)	11.8 (10.2–12.3)	0.253
Platelet (^*^10^3^)	172 (109–271)	118.5 (86–234)	0.576
PT	13.7 (12.7–21.1)	13.9 (11.7–21.8)	0.802
APTT	37.9 (30.8–59.2)	32.8 (27.7–47.6)	0.253
CRP (mg/dL)	8.5 (5.5–15.3)	11.5 (9.9–19.7)	0.219
GOT (U/L)	161.5 (86–575)	334.5 (111–431)	0.826
GPT (U/L)	45 (28–132)	71 (46–119)	0.743
Creatinine (mg/dL)	0.8 (0.54–1.59)	1 (0.59–1.69)	0.954
HCO3– (mmol/L)	16.5 (14.1–21.2)	13.9 (12.5–21.3)	0.658
Pre-ECMO troponin-I (ng/mL)	12.3 (5.4–29.6)	49.2 (4.1–98)	0.253
Pre-ECMO lactate (mg/dL)	31.1 (20–55.6)	98.6 (52.3–143)	0.001^*^
**Post-ECMO**			
Post-ECMO peak troponin-I (ng/mL)	19.3 (9.1–61.8)	85.3 (54.5–180.2)	0.013^*^
Post-ECMO peak CK-MB (ng/mL)	50.6 (29.7–140.4)	229.1 (29–300)	0.114
Post-ECMO creatinine (mg/dL)	0.7 (0.6–1.5)	1.2 (0.9–2.7)	0.068
Post-ECMO highest lactate (mg/dL)	33.8 (20.1–55)	101.9 (44–158)	0.018
Post-ECMO lowest lactate (mg/dL)	12.9 (10.3–17.5)	18.3 (12.6–22)	0.064
The incidence of acute kidney injury (%)	11 (47.8)	6 (60)	0.522
The need for hemodialysis (%)	4 (17.4)	5 (50)	0.053
The incidence of hypoxic encephalopathy (%)	1 (4.3)	5 (50)	0.008^*^
Intracranial hemorrhage (%)	0	2 (20)	0.15
LV venting (%)	9 (39.2)	3 (30)	0.617
**Outcome**			
Duration of ECMO (hours), median (IQR)	120 (95–201)	134 (67–187)	0.985
Days of hospitalization (days), median (IQR)	26 (21–57)	8 (6–12)	0.001^*^

*Statistical significance was set at p < 0.05. PRISM, Pediatric Risk of Mortality; ECRP, extracorporeal cardiopulmonary resuscitation; SBP, systolic blood pressure; DBP, diastolic blood pressure; ECMO, extracorporeal membrane oxygenation; LVEF, left ventricular ejection fraction; AV, atrioventricular; VT, ventricular tachycardia; PSVT, paroxysmal supraventricular tachycardia; PT, prothrombin time; APTT, activated partial thromboplastin time; GOT, glutamic-oxaloacetic transaminase; GPT, glutamic-pyruvic transaminase*.

* *Statistical significance was set at p < 0.05*.

### Cox Regression Analysis for In-hospital Mortality

Univariate Cox regression analysis demonstrated that higher levels of pre-ECMO lactate and post-ECMO lactate and lower post-ECMO LVEF were significantly associated with in-hospital mortality (Table 3). However, only a higher level of pre-ECMO lactate and lower post-ECMO LVEF remained as predictors of in-hospital mortality in the multivariate Cox analysis model.

**Table 3:** Univariate and multivariate Cox regression analyses for in-hospital mortality.

**Parameters**	**Univariate**		**Multivariate**	
	**Hazard ratio**	***p***	**Hazard ratio**	***p***
PRISM III score	1.083 (0.998–1.176)	0.056		
Pre-ECMO				
Pre-ECMO troponin-I (ng/mL)	1 (0.996–1.005)	0.835		
Pre-ECMO lactate (mg/dL)	1.027 (1.012–1.041)	<0.001^*^	1.023 (1.007–1.04)	0.005^*^
Pre-ECMO LVEF (%)	0.978 (0.932–1.027)	0.377		
Post-ECMO				
Post-ECMO peak troponin-I (ng/mL)	1.001 (0.998–1.005)	0.406		
Post-ECMO highest lactate (mg/dL)	1.017 (1.006–1.028)	0.002^*^		
Post-ECMO LVEF (%)	0.917 (0.875–0.962)	<0.001^*^	0.928 (0.883–0.976)	0.004^*^
The need for hemodialysis	4.329 (1.216–15.41)	0.024^*^		

* *Statistical significance was set at p < 0.05. PRISM: Pediatric Risk of Mortality; ECMO, extracorporeal membrane oxygenation; LVEF, left ventricular ejection fraction*.

### Test Characteristics Associated With In-hospital Mortality

**Figure 1:**
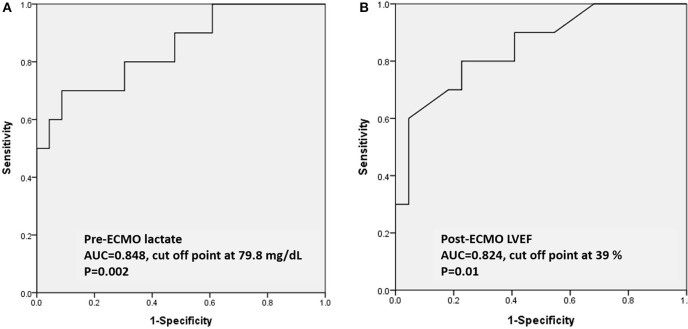
Based on the ROC curve analysis of pre-ECMO lactate and post-ECMO LVEF in predicting the survivors, the area under the ROC curve was 0.848 (95% CI, 0.697–0.999, *p* = 0.002) with a cutoff point of 79.8 mg/dL **(A)**; the area under the ROC curve was 0.824 (95% CI, 0.704–0.996, *p* = 0.01) with a cutoff point of 39% **(B)**. ROC: receiver operating characteristic; ECMO: extracorporeal membrane oxygenation.

**Figure 2:**
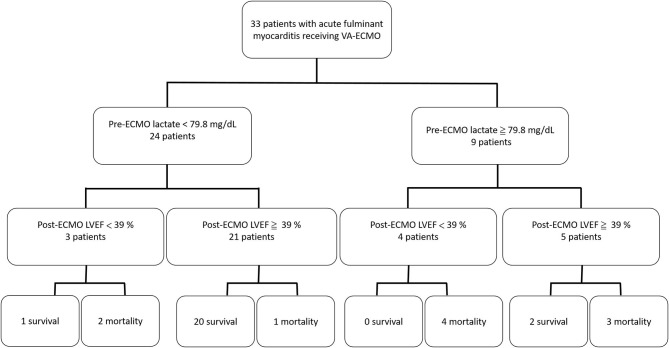
The Consort diagram with best predictive power of pre-ECMO lactate and post-ECMO LVEF for in-hospital mortality.

In ROC analysis to predict survival, the AUCs of pre-ECMO lactate, and post-ECMO LVEF were 0.848 (95% CI, 0.697–0.999, *p* = 0.002; Figure 1A) and 0.824 (95% CI, 0.704–0.996, *p* = 0.01; Figure 1B), respectively. The best cutoff values of the two factors are shown in Table 4. A pre-ECMO lactate level of 79.8 mg/dL and post-ECMO LVEF of 39% were appropriate cutoff points to predict mortality. Figure 2 showed the Consort diagram with best predictive power of pre-ECMO lactate and post-ECMO LVEF for in-hospital mortality.

**Table 4:** Best predictive power of pre-ECMO lactate and post-ECMO LVEF for in-hospital mortality.

**Parameter**	**Value**	**Sensitivity**	**Specificity**	**LR^**+**^**	**LR^**−**^**	**Youden index**
Pre-ECMO lactate (mg/dL)	79.8	0.7	0.91	8.1	0.33	0.61
Post-ECMO LVEF (%)	39	0.6	0.95	13.2	0.42	0.56

*ECMO, extracorporeal membrane oxygenation; LVEF, left ventricular ejection fraction; LR^+^, positive likelihood ratio; LR^−^, negative likelihood ratio*.

## Discussion

Acute fulminant myocarditis is a serious disease that progresses rapidly and the clinical course usually deteriorates within several hours. VA-ECMO is not curative therapy for acute fulminant myocarditis, but rather supports cardiopulmonary function to allow time for recovery of cardiac function or as a bridge to transplantation. Therefore, it is important to initiate ECMO support at the appropriate time in these patients. In this study, the survival rate of the children with acute fulminant myocarditis who received VA-ECMO was 69.2%, which is consistent with previous studies with reported survival rates ranging from 61 to 80% (12, 13, 21, 22).

Timely VA-ECMO implantation can improve the survival rate of patients with acute fulminant myocarditis to as high as 80% compared to those who do not receive VA-ECMO support, in whom the survival rate is only 50% (12, 23, 24). Therefore, the decision to initiate VA-ECMO should not be delayed due to the rapid deterioration in acute fulminant myocarditis. Previous studies have reported correlations between serum markers reflecting cardiac inflammatory status and end-organ function including troponin-I, liver enzymes, creatinine, and serum lactate with an aggressive disease process (12, 15). In the current study, we found that a pre-ECMO lactate level was the most powerful predictor of in-hospital mortality in the children with acute fulminant myocarditis receiving ECMO, and that the best cutoff value was 79.8 mg/dL. A higher lactate level can indicate decreasing cardiac output, and prolonged and worsening end-organ hypoperfusion in acute fulminant myocarditis. Therefore, an elevated lactate level may be the most important serum marker to prompt the early initiation of VA-ECMO to reverse shock and multi-organ dysfunction.

After VA-ECMO implantation, it is important to identify potential predictors of myocardial recovery which will affect the decision making for those who will not recover. Both troponin-I and LVEF were independent risk factors for mortality in this study, in which troponin-I represents myocardial injury and LVEF represents cardiac function. In addition, we identified that post-ECMO LVEF was the most powerful factor predicting recovery of myocardial function and mortality, with the best cutoff value of 39%. The post-ECMO LVEF improved early in the survivors in this study, and this improvement was sustained during follow-up, which is comparable with previous studies (12). However, for patients in whom post-ECMO LVEF does not improve, the use of left ventricular assist devices (LVADs) or urgent heart transplantation should be considered.

EMB is the golden standard to approve the diagnosis of myocarditis, but EMB has a low sensitivity and carries risks due to the invasive procedures. One study conducts at five centers reported that the EMB confirmed the diagnosis only in 41% of pediatric patients diagnosed with myocarditis and yielded a complication rate about 16% (25). The complication rate was especially higher in infant (30–40%) (25). One study reported that perforation of right ventricle was the most common critical complication in patients being assessing for myocarditis, requiring high dose of vasoactive-inotropic agents, or body weight <10 kg (26). In a study analyzing 514 pediatric patients diagnosed with myocarditis in American since 2006–2011, EMB was only performed in 26% patients (4), and most of those patients met the criteria of clinical diagnosis without receiving EMB (15). The presenting of prodromal illness was one important criteria for clinical diagnosis but not all patients finding the pathogen. Only 22.3% patients diagnosed with acute myocarditis in the Australian registry and 43% of the patients diagnosed with acute myocarditis in another study in American found the definite pathogen (27, 28). The patients in the current study all met the clinical diagnosis of acute myocarditis and the pathogen was reported in 51.5% of the patients.

### Limitations

The study has several limitations. First, the sample size was small, the study was retrospective, and it was conducted only at two pediatric centers. Therefore, there is risk of missing data and information bias. Second, EMB is the golden standard to confirm acute myocarditis. However, because EMB may cause complications in patients in a critical condition (16, 26), the procedure is not performed in pediatric patients at the two hospital. Therefore, the diagnosis of acute fulminant myocarditis in the two hospital is based on the criteria of “Probable acute myocarditis” as published in the Lancet in 2012 (24). Third, none of our patients received a LVAD or heart transplantation, because LVADs are very expensive and waiting times for heart transplants are very long in Taiwan. Hence, this study provides important information about which patients need to be evaluated for LVAD implantation or urgent heart transplantation.

## Conclusion

VA-ECMO can effectively improve the survival rate in children with acute fulminant myocarditis. Pre-ECMO lactate level was associated with mortality, with an optimal cutoff value of 79.8 mg/dL. After VA-ECMO implantation, post-ECMO LVEF was associated with mortality, with an optimal cutoff value of 39%. The use of LVADs or urgent heart transplantation should be considered if the post-ECMO LVEF does not improve.

## Data Availability Statement

The datasets generated for this study are available on request to the corresponding author.

## Ethics Statement

Because this was a retrospective study, the need for informed consent was waived from all participants in the study. This study was approved by the Institutional Review Board of Chang Gung Memorial Hospital (IRB Number: 201800094B0).

## Author Contributions

SH-H and H-PW: conceptualization. O-WC: methodology and software. C-YL and Y-TS: validation. Y-TS: formal analysis. Y-SC: investigation. H-TC: resources and data curation. E-PL and S-CC: writing—original draft preparation and writing—review and editing. S-CC: visualization and project administration. J-JL: supervision. All authors contributed to the article and approved the submitted version.

## Conflict of Interest

The authors declare that the research was conducted in the absence of any commercial or financial relationships that could be construed as a potential conflict of interest.
